# Altered motor control patterns in whiplash and chronic neck pain

**DOI:** 10.1186/1471-2474-9-90

**Published:** 2008-06-20

**Authors:** Astrid Woodhouse, Ottar Vasseljen

**Affiliations:** 1 Department of Public Health and General Practice, Faculty of Medicine, Norwegian University of Science and Technology (NTNU), N-7489 Trondheim, Norway

## Abstract

**Background:**

Persistent whiplash associated disorders (WAD) have been associated with alterations in kinesthetic sense and motor control. The evidence is however inconclusive, particularly for differences between WAD patients and patients with chronic non-traumatic neck pain. The aim of this study was to investigate motor control deficits in WAD compared to chronic non-traumatic neck pain and healthy controls in relation to cervical range of motion (ROM), conjunct motion, joint position error and ROM-variability.

**Methods:**

Participants (n = 173) were recruited to three groups: 59 patients with persistent WAD, 57 patients with chronic non-traumatic neck pain and 57 asymptomatic volunteers. A 3D motion tracking system (Fastrak) was used to record maximal range of motion in the three cardinal planes of the cervical spine (sagittal, frontal and horizontal), and concurrent motion in the two associated cardinal planes relative to each primary plane were used to express conjunct motion. Joint position error was registered as the difference in head positions before and after cervical rotations.

**Results:**

Reduced conjunct motion was found for WAD and chronic neck pain patients compared to asymptomatic subjects. This was most evident during cervical rotation. Reduced conjunct motion was not explained by current pain or by range of motion in the primary plane. Total conjunct motion during primary rotation was 13.9° (95% CI; 12.2–15.6) for the WAD group, 17.9° (95% CI; 16.1–19.6) for the chronic neck pain group and 25.9° (95% CI; 23.7–28.1) for the asymptomatic group. As expected, maximal cervical range of motion was significantly reduced among the WAD patients compared to both control groups. No group differences were found in maximal ROM-variability or joint position error.

**Conclusion:**

Altered movement patterns in the cervical spine were found for both pain groups, indicating changes in motor control strategies. The changes were not related to a history of neck trauma, nor to current pain, but more likely due to long-lasting pain. No group differences were found for kinaesthetic sense.

## Background

Whiplash associated disorders (WAD) have been studied mainly in comparison to asymptomatic subjects and it remains controversial whether WAD represent a diagnostic entity different from chronic neck pain. WAD patients are separated from chronic neck pain merely on the history of trauma [1]. The trauma designation is however not reflected in structural injuries of the cervical spine or the cerebrum [2,3]. Consequently, somatosensory and motor deficits have gained research attention. There are consistent findings of hypersensitivity and widespread pain in WAD compared to healthy subjects [4-7], and when comparing WAD patients to chronic neck pain patients [5,6,8]. While this points to centrally mediated somatosensory alterations in WAD, it is not clear whether motor areas are also affected. If such changes exist, altered motor control as well as kinaesthetic change should be present in WAD, which would also provide important information for clinicians.

There is conflicting evidence for presence of kinaesthetic change in WAD patients, as measured by head repositioning or joint position error (JPE) in comparison to healthy controls [9-14], and the reported repositioning errors are generally small, i.e. 2–5°. Non-traumatic chronic neck pain patients do not seem to have reduced kinaesthetic sense compared to healthy subjects [12,15,16]. The smooth pursuit eye movement test, believed to reflect cervical afferent dysfunction, has also shown inconsistent results [17-19].

There is consistent documentation for reduced standing balance and increased sway in WAD patients compared to healthy subjects [20-25]. Small differences, and only for difficult balance tasks, have been found between WAD and non-traumatic neck pain patients [23]. Less attention has been given to local motor control in the neck. Proprioceptive information from the neck greatly influence head and trunk position sense [26] and motor control [27] and mechanisms controlling head motion may be different from those that control the trunk [28]. WAD patients present with reduced cervical range of motion (ROM) relative to asymptomatic individuals [29,30], but they also show increased variability in ROM [31]. These tests may however be affected by motivational factors. For trajectory head movement patterns WAD patients showed greater variation between days than asymptomatic controls [32]. More jerky movement patterns were found in WAD and chronic neck pain patients compared to healthy subjects, but there were no differences between the two pain groups [31]. Although the latter two studies address relevant motor control issues, they were both small and neither could provide evidence for a difference between WAD and chronic non-traumatic neck pain. In fact, of the above studies on postural control only two included non-traumatic chronic neck pain patients in addition to WAD and healthy subjects [23,31]. In other words, the other studies were not designed to reveal whether postural control deficits are related to the trauma or just long-standing pain. If WAD patients are distinguished by altered motor control strategies, their movement patterns should be different from that seen in both non-traumatic neck pain patients and in healthy controls.

Pain has been shown to induce decreased variability in postural strategies in low back pain [33,34], and stiffening of spinal movements [33]. Cervical movements in the associated planes relative to the primary movement plane have been named conjunct motion, and might reflect protective postural control strategies. Conjunct motion was explored in this study to get an impression of motor function or "freedom of movement" in the neck. Conjunct motion at the *end *of primary range was investigated in a previous study which found small differences between WAD and healthy controls, but the study did not include non-traumatic neck pain patients [30]. In addition, movement deviations would more likely be found *during *execution of the primary motion which from a motor control perspective is more complicated.

In order to explore motor function characteristics of WAD patients in particular, control groups of non-traumatic neck patients and asymptomatic subjects were included in this study. The main purpose was to investigate motor control deficits in WAD compared to the two control groups in relation to conjunct motion, JPE, ROM and ROM variability.

## Methods

A case-control study with a total of 173 participants was conducted in the period Jan 2004 – Oct 2006. The study group consisted of persons with persistent WAD for more than 6 months (WAD group). Two control groups were included; a group of chronic non-traumatic neck pain patients (chronic neck pain group) and a group of asymptomatic volunteers (asymptomatic control group). All subjects gave written informed consent and the study was conducted in accordance with the Helsinki Declaration and approved by the Regional Ethics Committee.

### WAD group

Participants were recruited successively from WAD-patients referred to the National Center for Spinal Disorders, St Olav's Hospital, Trondheim, Norway. A total of 59 subjects with WAD injury classified as Québec Task Force grades I-II [35], were included. All suffered from neck pain and/or headache after a car collision where they had either been driver or passenger of a motor vehicle. Symptom duration of between 6 months and 10 years and onset of symptoms within 48 hours after the accident were criteria for inclusion. Subjects were excluded if they had WAD III-IV, had suffered a head injury during the accident or had surgery done in the cervical spine. They were also excluded if they had a history of similar symptoms previous to the accident or any known systemic disease that could explain their symptoms. Of the subjects in this group, 35 had an ongoing insurance compensation claim.

### Chronic neck pain group

A total of 57 subjects with chronic non-traumatic neck pain were recruited by local physiotherapists and general practitioners. Pain duration of at least 6 months and not more than 10 years was required for inclusion. Alternatively, the subjects could have repetitive episodes of pain of at least one week's duration every 3 months the last 2 years. Subjects were excluded if they had any history of neck trauma or any known systemic disease that could explain their symptoms.

### Asymptomatic control group

The asymptomatic control group included 57 subjects with no previous or current neck pain or history of neck trauma. Participants in this group were recruited from different workplaces and educational institutions.

The study was part of a more comprehensive study also involving diagnostic imaging of the cervical spine. Pregnant women and persons with contra-indications to MR imaging (e.g. pacemaker, magnetic aneurism clips, etc) were therefore excluded.

### Instrumentation

All cervical motion measurements were made using the 3 Space Fastrak (Polhemus, Inc, Colchester, Vermont, USA) with a sampling rate of 120 Hz. The system has been found to reliably record head position and cervical range of motion among asymptomatic persons as well as persistent neck pain patients [36-38]. The system includes a transmitter creating an electromagnetic field. The position and orientation of a sensor, in this study placed on the person's forehead with an elastic band is monitored by the transmitter as it moves in the electromagnetic field. The sensor gives the position and orientation of the head in relation to the transmitter and represents the measurements of the test-persons neck movements in three dimensions simultaneously. The Fastrak transmitter was placed on the upper part of the wooden backrest above the subjects' head. Custom-made Matlab-based software was used to store, interpret and quantify the data. The software included an algorithm which enabled the system to estimate the direction of a dominant axis of rotation during the movements. The two associated planes were then adjusted to the dominant primary plane axis. This was done by first estimating the average rotation matrix R_b of the sensor with respect to the transmitter throughout the recording, according to Stavdahl et al, 2005. The average orientation was subtracted from all rotation matrix samples R(t) in the recording, yielding an unbiased data set R_balanced(t) = R(t) * Transpose(R_b) with a zero mean (corresponding to the identity matrix). The dominant axis of rotation in this balanced set was subsequently estimated as the largest principal component of the aggregate rotation matrix R_Sigma = Sum_t (R_balanced(t)) (i.e. the arithmetic sum of all the sampled rotation matrices) [39]. The primary motion plane is given as the plane perpendicular to this principal component. In this way, movement planes were defined by the individual person's "choice" of direction during movement, as opposed to a fixed spatial direction of movement.

### Outcome variables

Maximal cervical ROM was recorded in the three primary motion planes. Conjunct motion in the two associated planes was recorded continuously throughout maximal primary range movements. The variability of maximal ROM has previously been used as a measure of sub-maximal effort in the testing situation due to fear of pain or movement. ROM-variability was in this study computed as the standard deviation (SD) of three repeated trials for each primary neck movement plane (rotation, side-bending and flexion/extension). JPE was recorded as the difference in head orientation at neutral position before and after cervical rotation. Neck pain and headache intensity at the time of testing was registered on numeric rating scales (NRS), where 0 denoted "no pain" and 10 "worst pain possible". Insurance compensation status in the WAD group was obtained from a self-reported questionnaire and used to explore differences within the group in pain intensity reports and ROM.

### Testing procedures

The examiner was not blinded to the subjects' group allocation but all commands were standardized. Maximal cervical range of motion was measured with the subject seated on a wooden bench with a footrest and a backrest. Shoulders and thorax were fixed to the backrest with shoulder straps when measuring maximal cervical spine movements. The subjects were asked to move "as far as possible" in all three primary motion planes: axial rotation, side-bending and flexion/extension. In each trial the movement was carried out in both directions of the plane with a short pause in the neutral position. Each movement trial was repeated 3 times. Movements were performed at a self-determined pace.

JPE was recorded with the subject comfortably seated with the head and neck in a neutral position with thorax/shoulders unfixed. A laser beam device was placed above the Fastrak sensor on the subject's head pointing to the middle of a target (bull's eye) approximately 1.5 meters in front of the subject's head to record neutral position. With the eyes closed, the subject was asked to rotate the head "as far as comfortable" towards the left, and then reposition the head to the starting position and give a verbal signal when he/she thought that the starting position was retrieved. The orientation (degrees) of the sensor at end-position was recorded. The subject was then asked to open his/her eyes and reorient the laser beam back to the middle of the target before the next trial. The procedure was repeated twice in each direction of cervical rotation.

### Data management and statistical analysis

**Figure 1:**
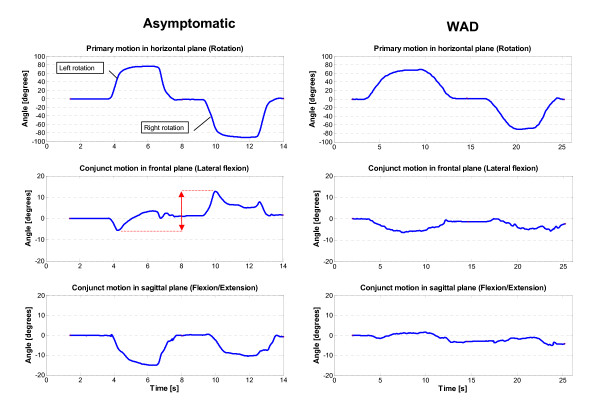
**Primary and conjunct motion recordings**. Movement plots during primary cervical rotation in the horizontal plane (upper panels) with the associated conjunct movements below. Cervical rotation was first carried out to the left then to the right in one sequence with a small pause in between (asterisks). Conjunct motion in side-bending (middle panels) and flexion/extension (lower panels) are shown for a healthy subject (left column) and a WAD patient (right column). The arrow in the left, middle panel illustrates the range of conjunct motion used for analyses. The sum of conjunct motion in the two associated planes was taken to express total conjunct motion for each primary motion. Note the stiffer movement pattern in the WAD patient (lower two panels to the right).

Maximal cervical ROM was calculated for the entire range of each primary movement plane by the mean and standard deviation (SD) of three trials. The SD of three repetitions of maximal ROM was used to express ROM-variability for each cardinal plane, and the mean value (SD_mean_) of the three planes was used in the analyses. Mean ROM in each primary plane was summed to express total cervical ROM. Conjunct motion was calculated as the maximum deviation range (°) in each associated conjunct motion plane during execution of the primary movement, i.e., the maximal ROM trials (Fig 1). The sum of conjunct motion in the two associated planes was taken to express total conjunct motion for each primary motion. JPE was registered in degrees as the absolute maximum repositioning error regardless of plane, i.e. the largest error value of the three planes was selected as the JPE. The mean JPE value from four test repetitions (two left, two right) was used for analysis.

Differences between groups in conjunct motion and maximal cervical ROM were analyzed by general linear modelling (GLM) and post-hoc by the Tukey-Kramer multiple comparison test. Conjunct motion was analyzed both with and without introduction of covariates and adjusted for age, gender, neck pain intensity on day of testing and range of motion in the primary plane. For some variables residual plots showed an increasing variance with increasing predicted values. Transformation of these variables using natural logarithms (Ln) gave acceptable residual plots and similar statistical results. Thus, the non-transformed values are reported. The Kruskall-Wallis test was used for evaluating group differences in JPE and ROM-variability. Differences in pain levels and ROM between subjects with and without ongoing compensation claims within the WAD group were evaluated by the two sample t-test. Group differences were considered significant at the p < 0.05 level. Analyses were performed using SPSS 14.0 and NCSS 2007 (Utah, USA).

## Results

Of the 173 subjects participating in the study, motion data from three persons in the WAD group were lost due to technical problems during data collection. In addition, one person in the WAD group did not report compensation status. Baseline characteristics of the three groups are given in Table 1. There was no significant gender difference between groups, but the chronic neck pain group was on average 5.7 yrs (p < 0.001) and 5.3 yrs (p < 0.001) older than the WAD and the asymptomatic groups, respectively. The mean intensity levels of head and neck pain were higher in the WAD group compared to the control groups (Table 1).

**Table 1:** Subject characteristics, neck pain and headache intensity

	**WAD ***n *= 56	**Chronic Neck pain ***n *= 57	**Asympt ***n *= 57	*p*^(3)^
Gender (Female/male)	34/22	38/19	28/29	0.16
Age (yrs)^1)^	38.19(10.8)	43.7(12.6)	38.2(10.9)	0.01
Age range (yrs)	20–61	20–65	21–61	
Pain levels				
Neck pain^1) ^(0–10)^2)^	5.60 (2.49)	3.84 (1.74)	0.04 (0.19)	< 0.01
Headache^1) ^(0–10)^2)^	4.73 (2.85)	2.05 (2.38)	0.15 (0.59)	< 0.01

^1) ^Data are means (SD), ^2) ^0; no pain/10; worst imaginable pain, ^3) ^Overall group differences.

There were no differences within the WAD group between subjects with (n = 35) and without ongoing insurance compensation claims, although small non-significant reduction in ROM and higher mean intensity levels of headache and neck pain were found in those with claims (Table 2).

**Table 2:** Pain intensity and total ROM among WAD patients with and without ongoing insurance compensation claims. Given values are mean (SD)

	**Ongoing Compensation ***n *= 35	**Not ongoing Compensation ***n *= 20	*p*
Neck pain (0–10)	5.66 (2.65)	5.50 (2.26)	0.824
Headache (0–10)	5.03 (2.85)	4.20 (2.84)	0.304
Total ROM (°)	246.19 (88.51)	264.45 (64.32)	0.752

### Conjunct motion

**Figure 2:**
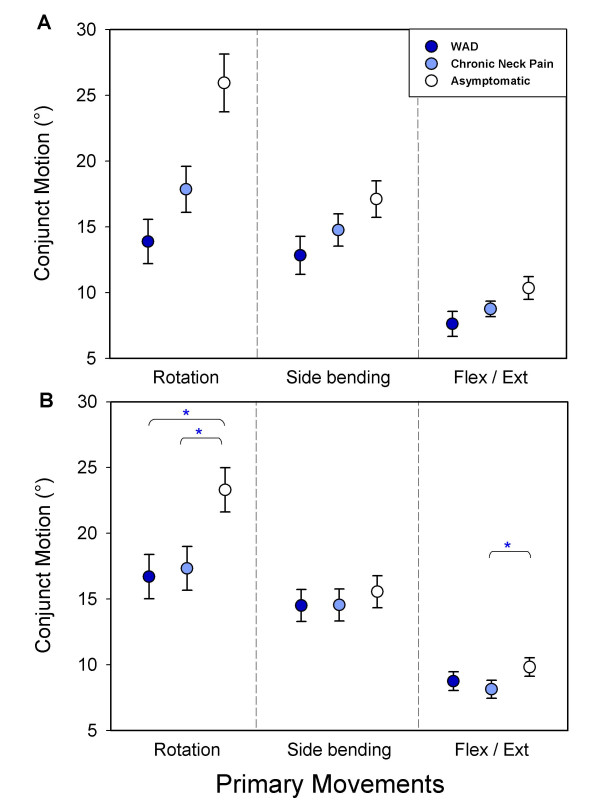
**Conjunct motion during primary cervical rotation**. Total conjunct motion for each primary cervical motion plane and group. The primary motions are denoted on the figure. Symbols illustrate mean range of total conjunct motion with 95% confidence intervals in the three study groups (A). The data in (B) are adjusted values for group differences in age, gender, current neck pain and primary range of motion. Significant group differences are denoted only in (B).

Conjunct motion was largest during primary cervical rotation and smallest during primary flexion/extension for all study groups (Fig 2). Reduced conjunct motion in the pain groups was found on most variables in the unadjusted analyzes, and consistently between WAD and healthy subjects (Table 3). A number of differences disappeared after adjusting for age, gender and current neck pain intensity, and adding range of motion in the primary plane as a covariate resulted in significant difference only between the pain groups and the asymptomatic subjects, and only for conjunct side-bending during primary rotation (Table 3).

**Table 3:** Conjunct motion range and group comparisons. Values (°) are in mean (SD).

	**WAD ***n *= 56	**Chronic Neck Pain ***n *= 57	**Asympt**. *n *= 57	***p*^(1)^**	***p***^(2)^	***p***^(3)^
**Primary Rotation**						
Conjunct Side-bending	7.00 (3.52)	8.87 (4.09)	13.15 (4.08)	a **	a **	a **
				b *	b *	
				c **	c **	c **
Conjunct Flexion/Extension	6.89 (3.22)	8.99 (3.26)	12.79 (5.23)	a **	a **	
				b **	b *	
				c **	c *	
						
**Primary Side-bending**						
Conjunct Rotation	6.24 (2.91)	7.19 (2.18)	8.68 (2.97)	a **		
				c **		
Conjunct Flexion/Extension	6.59 (2.98)	7.57 (3.12)	8.43 (2.69)	a **		
						
**Primary Flexion/Extension**						
Conjunct Rotation	3.95 (1.89)	4.58 (1.32)	5.10 (1.53)	a **		
				b*		
Conjunct Side-bending	3.66 (1.82)	4.18 (1.24)	5.24 (2.26)	a **	a *	
				c **		

^1) ^Adjusted for age and gender. ^2) ^Adjusted for age, gender and current neck pain intensity. ^3) ^Adjusted for age, gender, current neck pain intensity and range of motion in the primary plane. * p < 0.05, ** p < 0.01, a = WAD vs Asymptomatic, b = WAD vs Chronic Neck Pain, c = Chronic Neck Pain vs Asymptomatic

Group differences in total conjunct motion were most clearly seen during primary rotation, and separated the three groups significantly (p < 0.01), (Fig 2A). Total conjunct motion during primary rotation was 13.9° (95% CI; 12.2–15.6) for the WAD group, 17.9° (95% CI; 16.1–19.6) for the chronic neck pain group and 25.9° (95% CI; 23.7–28.1) for the asymptomatic group (Fig 2A). Importantly, group differences in total conjunct motion during primary rotation were significant only between the pain groups and the asymptomatic subjects after controlling for age, gender, current neck pain and range of motion in the primary plane (p < 0.01), and the difference between the two pain groups disappeared, p = 0.60 (Fig 2B). The adjusted mean values were 16.7° (95% CI; 15.0–18.4), 17.3° (95% CI; 15.7–19.0), 23.3 (95% CI; 21.6–25.0) in the WAD, chronic pain and asymptomatic groups, respectively. A small difference remained between chronic neck pain and the asymptomatic groups for total conjunct motion during primary flexion/extension (p < 0.01).

### Cervical range of motion, ROM variability and JPE

Maximal primary cervical motion ranges, total cervical ROM, ROM-variability and JPE for the three study groups are shown in Table 4. The WAD group had significantly less ROM than the two control groups in all three primary motion planes as well as total cervical ROM. The differences remained significant even after adjusting for age, gender and pain intensity (p < 0.01). Also the chronic neck pain patients had significantly less motion in all primary planes compared to the asymptomatic controls. There were no significant group differences in ROM-variability (SD_mean_) or JPE.

**Table 4:** Maximal cervical ROM, ROM-variability and JPE. Values (°) are mean (SD).

	**WAD ***n*= 56	**Chronic Neck Pain ***n*= 57	**Asympt**. *n*= 57	*p*^(1)^
Rotation	106.2 (34.7)	133.1 (18.6)	151.7 (13.5)	< 0.01
Side-bending	60.9 (18.8)	72.2 (13.2)	84.9 (13.8)	< 0.01
Flexion/Extension	81.8 (34.6)	114.0 (20.0)	134.0 (20.7)	< 0.01
Total cervical ROM	248.9 (83.7)	319.4 (44.1)	370.7 (43.1)	< 0.01
				
ROM Variability (SD_mean_)	3.55 (1.73)	3.48 (1.70)	3.27 (1.28)	0.82
JPE	3.35 (1.6)	3.17 (1.1)	2.86 (1.2)	0.30

^1) ^Overall group differences

## Discussion

The main finding in this study was that patients with chronic neck pain showed altered motor control in the cervical spine. We found no indications for differential effects between traumatic and non-traumatic chronic neck patients in terms of conjunct motion. No group differences were found in ROM-variability or joint position error. Maximal cervical range of motion in the primary planes was however significantly reduced among the WAD patients compared to both control groups.

Conjunct motion was included in this study to get an impression of motor function or motion stiffness during execution of primary plane movements, believed to reflect central nervous motor control strategies [40,41]. Conjunct motion in the cervical spine has been studied in asymptomatic subjects [42-45], but only two studies have included WAD and healthy subjects [30,46]. Marginal reductions in conjunct motion in the WAD group were observed in both studies. A limitation in the study by Dall'Alba et al was that conjunct motion was recorded only at end of primary motion. In our study, conjunct motion was recorded during primary movements and is to our knowledge the first to compare WAD patients to both non-traumatic neck pain and healthy subjects. The study groups were separated on most conjunct motion measures in the unadjusted analyses (Tab. 3). It is possible that conjunct motion mainly happens towards end of physiological range and is therefore reduced due to reduced primary ROM. However, differences between the pain groups and the healthy controls remained after controlling for ROM in the analysis. Neither could the differences between the pain groups and healthy subjects be explained by current neck pain intensity. This indicates that stiffer or more guarded movement patterns are a response to long-standing pain. This response was irrespective of a trauma history. For all groups the largest conjunct motion ranges were found during primary cervical rotation (horizontal plane) and, interestingly, stiffer movement patterns in the pain groups were also most evident during cervical rotation. This complies with a general understanding that cervical rotation demands more complex physiological coupled motions due to the anatomic structures, such as the orientation of cervical zygapophysial and uncovertebral joints [47]. Changes in motor control due to chronic pain may thus be observable only in more complex movement patterns.

Whether reduced conjunct motion reflects altered motor control strategies needs further investigation. If confirmed, it remains to reveal if such changes are peripherally driven or merely confined to central processing mechanisms. Perceived position of the head is greatly influenced by proprioceptive signals from the neck [26]. Hypothetically, cervical microtrauma involving mechanoreceptors may cause corrupted afferent information and sensorimotor mismatch [48]. The head relocation test, equivalent to the JPE test, was introduced to detect alterations in cervical proprioception [49]. We thus expected that disturbances in proprioception would be reflected in the JPE test, but we found no difference between the groups. Consequently, proprioceptive dysfunction gives a poor explanation for the observed alterations in conjunct motion in the pain groups. Since current pain intensity could neither account for reduced conjunct motion, it may be argued that the observed changes in motor control reflect central nervous motor processing adaptations due to long-standing pain rather than disturbances in concurrent afferent inputs. Musculoskeletal pain has been reported to induce cortical reorganization in both somatosensory and motor areas [40]. Maladaptive cortical sensimotor integration with chronic neck pain has been suggested [50] and neuromagnetic imaging has shown altered responses in the motor cortex due to chronic pain [51]. Although local trauma to mechanoreceptors in the neck cannot be excluded, our findings favour centrally driven alterations in motor control due to long-standing pain.

Kinaesthetic sense deficits in patients with cervical pain have in previous studies been demonstrated as reduced accuracy, or increased cervical joint position error, when relocating the head to the initial position after a maximal or sub-maximal cervical movement [9,11,14,49,52,53]. The evidence is inconclusive, however, as other studies have found only small or no differences in JPE among neck pain patients [13,15,22,54] or greater JPE only among those with the highest levels of pain and disability [10]. Various procedures have been used to record JPE of the cervical spine and the variation in set-up may account for the conflicting findings. In our study, group differences in JPE were very small (<1°) and statistically non-significant.

As expected, and in agreement with other studies [29,30,55,56], primary ROM differentiated between WAD patients and healthy controls also in this study. The WAD patients also had significantly less cervical ROM compared to the chronic neck pain group, even after adjusting for current pain intensity, gender and age. Although ROM differentiated between the two groups of neck pain patients, the diagnostic value of ROM-testing can be questioned. Patients could intentionally simulate less motion for beneficial reasons in cases of unclarified insurance compensation. However, the subgroup of WAD-patients with ongoing compensation claims in this study performed similarly to the rest of the WAD group, which leaves no suspicion of simulation of abnormal motion.

ROM-variability from repeated recordings of maximal cervical range has been introduced as a measure that could distinguish between maximal and sub-maximal effort in the ROM testing situation [57,58]. Hypothetically, both simulation of ROM and pain-related fear of movement may explain a sub-maximal effort. Sub-maximal effort may presumably increase variability between repeated trials of maximal ROM. This is due to an assumption that physiological end-of-range in the cervical spine is not reached with a sub-maximal effort, resulting in greater variability in endpoints of the repeated movements. Two studies have reported larger ROM-variability among WAD patients compared to healthy controls [31,56]. In our study, no increase in ROM-variability among WAD patients or chronic neck pain patients was found. However, the use of SD_mean _as measure of variability implies that the "size" of ROM did not influence the variability measure. An alternative that would normalize the variation to the mean ROM is the coefficient of variation (CV). However, the small variability of the nominator (SD_mean_) relative to the large variability in the denominator (mean ROM) would make the CV heavily depend on the latter and thus add little information beyond that of mean ROM. A correlation analysis was performed between mean ROM and SD_mean _to investigate whether SD_mean _was dependent on the "size" of mean ROM and thus needed to be normalized. No correlation was found in any of the study groups, and SD_mean _was considered the best variability measure. When direction-specific maximal ROM is repeatedly recorded from neutral position, variability in the neutral position starting point could hypothetically add to the variability in ROM. This factor was eliminated by recording total plane ROM in our study. Since increased ROM-variability was not found between any of the groups, sub-maximal effort due to simulation, pain or fear of pain was not confirmed in this study.

## Conclusion

Compared to asymptomatic controls both whiplash and chronic neck pain patients showed reduced conjunct motion, particularly during primary cervical rotation. We found no indications for a difference between traumatic and non-traumatic neck pain patients. Reduced conjunct motion was not explained by variation in current neck pain or range of motion in the primary plane. Less flexible movement patterns in the two pain groups compared to the asymptomatic group may reflect motor control adaptations, possibly due to long lasting pain. JPE and maximal ROM-variability did not separate the study groups. The whiplash patients showed reduced maximal cervical ROM compared to both control groups, and the chronic neck pain group had less ROM compared to the asymptomatic controls. No significant differences in ROM and pain intensity were found between WAD-patients with ongoing insurance compensation claims and the rest of the WAD-group.

## Competing interests

The authors declare that they have no competing interests.

## Authors' contributions

The authors have contributed equally to the conduction of the study, the data analysis and the writing of the manuscript. The final manuscript has been read and approved by both authors.

## Pre-publication history

The pre-publication history for this paper can be accessed here:


